# The Impact of Flooding on Snail Spread: The Case of Endemic Schistosomiasis Areas in Jiangxi Province, China

**DOI:** 10.3390/tropicalmed8050259

**Published:** 2023-04-30

**Authors:** Shang-Biao Lv, Ting-Ting He, Fei Hu, Yi-Feng Li, Min Yuan, Jing-Zi Xie, Zong-Guang Li, Shi-Zhu Li, Dan-Dan Lin

**Affiliations:** 1 Jiangxi Provincial Institute of Parasitic Diseases, Nanchang 330096, China; 2 National Institute of Parasitic Diseases, China CDC (Chinese Center for Tropical Diseases Research), Key Laboratory on Parasite and Vector Biology, National Health Commission, WHO Collaborating Centre for Tropical Diseases, National Center for International Research on Tropical Diseases, Ministry of Science and Technology, Shanghai 200025, China; 3 Jiangxi Province Key Laboratory of Schistosomiasis Prevention and Control, Nanchang 330096, China

**Keywords:** flood, snail spread, schistosomiasis, Jiangxi province

## Abstract

Flooding is the main natural factor in snail diffusion, and it has a negative impact on schistosomiasis transmission. There are few studies on the spread and migration of snails following a flood; therefore, we aimed to investigate the influence of flooding on snail diffusion and explore the characteristics and laws of snail diffusion in Jiangxi Province. By using a retrospective survey and cross-sectional survey, the data on snail spreading in Jiangxi Province from 2017 to 2021 were collected. The distribution, nature, and area of snail spread were systematically analyzed in combination with the hydrological situation, types of region, and types of flood. From 2017 to 2021, a total of 120 snail-spread environments were found, including in 92 hilly areas and in 28 lake areas. The areas caused by flood and by other means numbered 6 and 114, respectively. The proportions of recurrence, expansion, and first-time occurrences were 43.42%, 38.16%, and 18.42%, respectively, and the 14 new snail environments were only distributed in the hilly areas. With the exception of 2018, the ratio of snail-spread areas in the hilly region was higher than that in lake region in other years. The average density of live snails was 0.0184–1.6617 no./0.1 m^2^ and 0.0028–0.2182 no./0.1 m^2^ in the hilly region. Among the 114 environments affected by floods, 86 consisted of hilly environments, including 66 spreading environments affected by rainstorm floods, and 20 rainstorm debris flow environments. There were 28 lake areas, of which 10 were in the Jiangxi section of Yangtze River and were affected by rainstorm floods. Snail spread following flooding has a certain ‘lag effect,’ and = simple annual changes in hydrological characteristics have little effect on the diffusion of snails or on their density = in the affected environment, but it is more closely related to local floods. The hilly environments are more susceptible to floods than the lake region, and the risk of snail spread is much higher in the hilly than in the lake region.

## 1. Introduction

Oncomelania hupensis is the only intermediate host of Schistosoma japonicum in China. Where schistosomiasis is endemic, Oncomelania hupensis is found in abundance. Snail control is one of the effective measures to block the transmission of or eliminate schistosomiasis [1,2,3]. In China, according to the geographical environment, snail habitats are classified into three types, lakes, hills, and water networks, and these are mainly distributed in the marshland in the middle and lower reaches of Yangtze River, the marshland of Dongting Lake and Poyang Lake, and in hilly ditches, fields, ponds, and other environments in Yunnan, Sichuan, Guangxi, and Zhejiang [4]. Over the past 70 years, various measures, such as molluscicides, environmental modifications, and integrated management, have been used to control snail populations, and the rate of schistosomiasis epidemics has dropped to its lowest levels in history in snail-infested areas. The size of snail-infested areas has decreased from 14.8 billion square meters when schistosomiasis control first began to 3.69 billion square meters in 2021, a decrease of 97.56%. Among the regions affected, the lake, hilly, and water-network regions were 3497.37 km^2^, 191.81 km^2^, and 3.50 km^2^ in size, respectively [5,6]. Although the snail areas have been effectively controlled, new and reemerging environments develop every year in endemic schistosomiasis provinces across the country. From 2017 to 2021, the total size of newly infested snail areas nationwide reached 2571.77 km^2^, of which five lake-region provinces accounted for 98.79% [7,8,9]. In addition, from 2009 to 2017, a total of 59.9 million m^2^ of new snail infestations were found downstream of the Three Gorges dam, accounting for 93.70% of the total newly infested snail area [10,11,12,13].

Oncomelania hupensis is a mollusk, with a distribution, reproduction, and growth that are influenced by natural factors, such as floods, climate change, environmental changes, and human and animal activities. Social factors, including water-conservation projects, flood control, levee construction, wetland protection, and farming methods, also influence their distribution, reproduction, and growth [4]. Water is one of the necessary conditions for snail growth and reproduction. Locations with fluctuating water levels or slow water flow and vegetation growth are often suitable habitats for snails. Studies have shown that changes in water levels greatly affect the distribution of oncomelanid snails, and floods are extreme expressions of water level changes [14]. 

Jiangxi Province used to be one of the provinces in China with a relatively severe prevalence of schistosomiasis. In 2015, the province reached a standard level of control over the transmission of schistosomiasis. As of the end of 2021, twenty-four out of thirty-nine counties in the province where schistosomiasis is prevalent had reached an elimination standard, six counties had reached a transmission-interrupted standard, and nine counties had maintained transmission-control standards [11]. However, because the snail-breeding environment has not been completely changed, especially the water situation of winter land and summer water in the Poyang Lake area, infestations are difficult to control artificially; snails still breed, reproduce, and spread in the Poyang Lake area, along the beaches of Yangtze River, and in some hilly ditches, wasteland, and paddy fields in northeast and central Jiangxi. The snail areas in Jiangxi Province have always ranked second in the whole country, reaching 849.38 km^2^, 23.00% of China’s total infested area, with the snail habitats divided into hilly and lake types. In recent years, the snail area in Jiangxi Province has been on the rise [6,15,16]. It is obvious that the breeding, reproduction, and spread of snails are increasingly becoming a huge risk in schistosomiasis control.

In China, numerous studies have shown that floods have a negative impact on schistosomiasis transmission. However, there are few studies that use the characteristics of floods as complex systems to analyze the spread and migration of snails [17,18,19,20]. Therefore, in this paper, the distribution, nature and areas of snail diffusion in Jiangxi Province from 2017 to 2021 were systematically analyzed in combination with the hydrological situation, type of region, and flood invasion methods, in order to explore the characteristics of snail spread in Jiangxi Province and provide a scientific basis for the formulation of snail-control measures in Jiangxi Province and even the whole country.

## 2. Method 

### 2.1. Snail Spreading

#### 2.1.1. Data Source

Based on the snail-census results in 2016 [16], the snail diffusion in historical snail environments or in snail-free environments adjacent to snail-occupied environments in endemic schistosomiasis areas over the whole province was investigated. A combination of retrospective and current situation surveys was used to review and collect the investigation data of snails in endemic schistosomiasis counties from 2017 to 2020. Additionally, to analyze the impact of the catastrophic flood disaster in Jiangxi province in 2020 on snail spread in endemic schistosomiasis areas, a special snail-diffusion survey was carried out in the affected endemic areas in the spring of 2021, and the area surveyed was suspected to be a snail-breeding environment and a historical snail environment affected by floods. The snail survey was conducted according to the industry-standard survey of oncomelanid snails (WS/T563-2017) using a snail frame measuring 0.1 m^2^, according to the standard specified by the Ministry of Health in China. 

#### 2.1.2. Spreading-Environment Types

In accordance with snails’ natural habits, the spreading environment used in the study was divided into a hilly region and a lake region, and the snail indicators, such as the snail area, average density of live snails, and their occurrence rate (%) in snail frames were counted for each spreading environment.

#### 2.1.3. Spreading Properties

Based on the results of the snail-proliferation survey and historical information, the diffusion properties were divided into expansion environment (expansion), recurrence environment (recurrence), and new environment (new). The expansion environment is an environment in which the snail area is expanded due to natural factors in the snail-free environment, to which the original snail distribution is connected. The recurrence environment is an environment in which snails are confirmed to be extinct after successful control in a historical snail environment, and live snails have been found again several years later. The newly discovered snail environment refers to the environment where snails are detected for the first time in an environment in which they had not been observed before.

### 2.2. Flooding

#### 2.2.1. Water Information

Checking the water-resources bulletin of Jiangxi Province, water information, such as hydrological data of the Poyang Lake basin, annual rainfall of the province, and the extent of flooding in endemic schistosomiasis areas since 2016 were collected. According to the annual precipitation or annual runoff, the annual hydrological characteristics from 2016 to 2021 are classified as wet year, median-water year, or dry year [21,22,23].

#### 2.2.2. Flood-Invasion Manner

Based on the local flooding data, the way in which each snail-spread environment was flooded was divided into the following manner [24,25].

Storm-flood type: A flood formed by heavy rain fall with a greater-than-normal intensity, through the production and confluence of flows in ditches, rivers, and other streams. The main characteristics of the flood are the high peak volume, long duration, and wide flooding area.

Storm-debris-flow type: A flood that contains a large amount of sediment, clay, gravel, rock, and other types of solid material in hilly areas, which are mixed with stormwater to make the gully area move or flow and slide slowly down the slope of the gully.

Submerged-lake type: A flood causing the water level of Poyang Lake to rise rapidly due to the joint influence of the top backflow of Yangtze River and the flooding of Ganjiang, Fei, Xinjiang, Rao, and Xiu Rivers into Poyang Lake, causing the beach of Poyang Lake to become inundated by the flood for a long period of time.

### 2.3. Data Analysis

Two snail indicators, the amount of space occupied by live snails in the snail frame and calculated as percentage of frames with live snails and the mean density of live snails, were studied using a descriptive statistical analysis. The qualitative data were compared using a chi-squared test (χ^2^) test and the quantitative data were analyzed using a variance analysis. A linear regression analysis was used to compare the snail-diffusion-area ratio and annual change trend. All data were statistically analyzed with SPSS 20.0 software (IBM, Armonk, NY, USA). Any *p* < 0.05 indicated that the difference was statistically significant.

$$\begin{array}{c} \mathrm{Percentage}\;\mathrm{of}\;\mathrm{frames}\;\mathrm{with}\;\mathrm{live}\;\mathrm{snail}\;\left(\%\right)=\frac{\mathrm{frames}\;\mathrm{of}\;\mathrm{live}\;\mathrm{snails}}{\mathrm{Number}\;\mathrm{of}\;\mathrm{investigation}\;\mathrm{frames}}\times 100\%\\ \mathrm{Mean}\;\mathrm{density}\;\mathrm{of}\;\mathrm{live}\;\mathrm{snail}\;\left(\mathrm{No}./0.1m^{2}\right)=\frac{\mathrm{number}\;\mathrm{of}\;\mathrm{live}\;\mathrm{snails}}{\mathrm{Number}\;\mathrm{of}\;\mathrm{investigation}\;\mathrm{frames}} \end{array}$$

## 3. Results

### 3.1. Analysis of the Oncomelania Snail Spread

**Figure 1:**
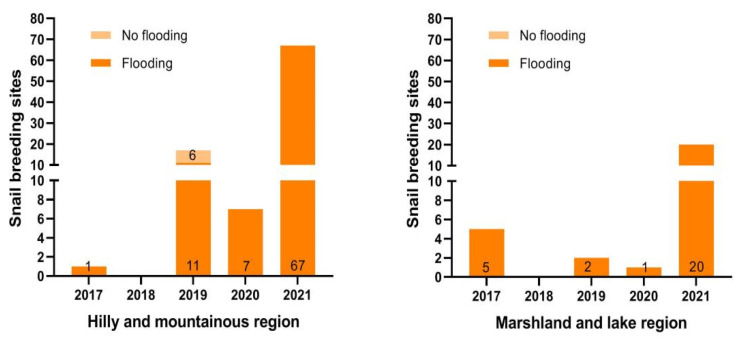
The annual distribution of the number of snail-spread environments in different endemic areas.

From 2017 to 2021, a total of 120 snail-spread environments were found, with varying degrees of occurrence in all years except 2018. Among these, the numbers of hilly and marshland regions were ninety-two and twenty-eight, respectively, of which six were not caused by floods. There was a significant difference in the number of snail-spread environments between the flooded and non-flooded regions (χ^2^ = 194.40, *p* = 0.00). In addition, the number of spreading environments in the hilly areas was greater than that in the lake region for all years except 2017 (Figure 1).

#### 3.1.1. Distribution of the Snail-Spread Environment

The 114 snail-spread environments caused by floods were mainly distributed in the hilly areas of northeastern Jiangxi, followed by Yangtze River and Poyang Lake. In particular, six environments were distributed in the hilly area of northeastern Jiangxi and Yangtze River in 2017, thirteen and eight environments were distributed in the hilly areas of northeastern Jiangxi and the river channel of Poyang Lake in 2019 and 2020, respectively, and eighty-seven environments were distributed in the hilly areas of northeastern Jiangxi, Yangtze River, and Poyang Lake in 2021. Furthermore, the beach elevation of Poyang Lake was 16.1–17.9 m (Wusong benchmark), and the beach elevation in the estuary of the Poyang Lake inlet was 10.0–12.5 m.

#### 3.1.2. Snail-Spread Area

The spread-area results from 2017–2021 showed that the hilly areas’ spread ratio was greater than that of the lake region in each year except for 2018, and that it increased from year to year (R^2^ = 0.854, F = 17.615, *p* = 0.025). In the lake region, the area ratio remained generally stable from 2017 to 2020, and increased rapidly by 1.49% in 2021, but there was no significant trend from year to year (R^2^ = 0.51, F = 3.12, *p* = 0.18) (Table 1).

#### 3.1.3. Snails’ Spreading Properties 

In 2017–2021, the proportions of snail recurrence, expansion, and first-time occurrences were 43.42%, 38.16%, and 18.42%, respectively. A pairwise comparison showed that the recurrence rate was significantly higher than the first-time-occurrence rate (χ^2^ = 11.119, *p* = 0.001), and was not statistically different from the expansion rate (χ^2^ = 0.436, *p* = 0.509). The expansion was significantly higher than the first-time occurrence (χ^2^ = 7.297, *p* = 0.007). Snail recurrence and expansion were present in both hilly and lake environments, and the 14 new environments were only found in the hilly region. The mean density of live snails was highest in the expansion environment, followed by the new environment and, next, by the recurring environment, while the percentage equating to the amount of space occupied by live snails in the snail frames was highest in the new environment, followed by the expansion environment and the recurrence (Table 2).

#### 3.1.4. Snail Situation 

The mean density of live snails in the spreading environments in the hilly and lake regions was 0.018–1.66 no./0.1 m^2^ and 0.0028–0.22 no./0.1 m^2^, respectively, with a median value of 0.58 no./0.1 m for the hilly region, which was significantly higher than the median value of 0.039 no./0.1 m for the lake region (*p* = 0.00). 

**Figure 2:**
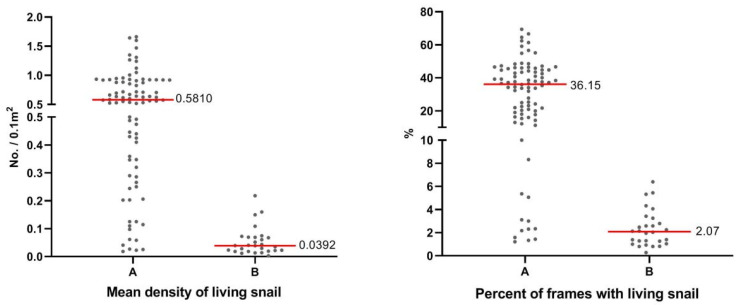
The Oncomelania hupensis’ situation in the diffusion environments of different endemic regions.

The space occupied by live snails in the frames was 1.21–69.44% and 0.28–6.40% in the hilly and lake regions, respectively. The median percentage of the frames with snails in the hilly region was 36.15%, which was significantly higher than that of the lake region (2.07%) (*p* = 0.000) (Figure 2).

A refers to the hilly and mountainous regions and B refers to the marshland and lake regions.

### 3.2. Impact of the Flooding Patterns on the Spread of Snails

The annual-rainfall-analysis results showed that 2016 and 2019 were wet years, 2017 and 2020 were median water years, and 2018 was a dry year. Regardless of the type of year, disasters with different degrees of flooding occur in some areas of Jiangxi Province every year. From 2016 to 2019, the flood disasters in the endemic area were mainly concentrated in the hilly areas of northeastern Jiangxi and the Jiangxi section of Yangtze River, and only short-term flooding above the warning level occurred in Poyang Lake. In July 2020, the Poyang Lake area was doubly affected by the flood’s top-down irrigation from the five rivers and Yangtze River, which was hit by the catastrophic flood in 1998. It rose at a rate of more than 0.4 m for 8 consecutive days, with a maximum daily increase of 0.65 m, varying by nearly 7 m from its lowest to its highest. The water levels at the Xingzi, Poyang, Yongxiu, and Guxiandu hydrological stations exceeded the historical water levels by 0.11 m, 0.14 m, 0.15 m, and 0.25 m, respectively. All 185 single retreat dikes in the Poyang Lake area were used for flood diversion and inflow for the first time since they were built, in 2007, on July 11, and the historical or suspected snail environment in the flood-detention basin was submerged (Table 3).

Among the 114 environments affected by flooding, there were 86 diffuse environments in the hilly areas, including 66 spreading environments affected by storm flooding and 20 by storm-debris flow. There were twenty-eight lake and marsh areas, of which ten areas in the Jiangxi section of Yangtze River were affected by storm flooding, fifteen were affected in the main lake area of Poyang Lake, and three were affected in the river channel, which was affected by the lake inundation.

According to the flood-invasion types, the average density of live snails and the percentage of occupied space in the frames with live snails, from high to low, were the rainstorm-flood type (0.50 No./0.1 m^2^ and 22.84 %), rainstorm-debris-flow type (0.33 No./0.1 m^2^ and 14.31%), and submerged-lake type (0.06 No./0.1 m^2^ and 2.16%). There were significant differences in the percentages of occupied space in the frames with live snails between the two groups (χ^2^ = 206.97, 2668.38, 1001.83, P_all_ = 0.00). However, the differences in the mean density of live snails were only statistically significant between the rainstorm-flooding and submerged-lake types (F = 20.60, *p* = 0.00). There was no significant difference between the rainstorm-flood type and the rainstorm-debris-flow type, nor between the rainstorm-debris-flow type and the submerged-lake type (F = 1.38, 27.73; *p* = 0.339, 0.06) (Table 4).

## 4. Discussion

The “Healthy China 2030” planning outline proposes that the whole country will achieve the goal of eliminating schistosomiasis by 2030 [26]. *Oncomelania hupensis* is the only intermediate host of Schistosoma japonicum in China, and controlling the snail population plays a vital role in blocking and eliminating schistosomiasis. Jiangxi Province is one of the provinces with a severe schistosomiasis prevalence and is also one of the main provinces with frequent flooding in China. At present, the snail areas in Jiangxi Province cover 849.38 km^2^, and they are mainly distributed in Poyang Lake, along Yangtze River, and in the beach environments to which the water system is connected, as well as in low-lying, slow-flowing, and overgrown irrigation ditches and paddy fields, dry land, ponds, weirs, hillside wasteland, and other environments among the hills in the northeast and central–southwest areas of Jiangxi [16]. Numerous studies have shown that flood disasters are one of the important factors causing the resurgence of snail populations [26,27,28]. The results of this study also verified this point. The number of diffusion environments caused by floods was 12.67 times that of non-floods.

The results showed that 75.44% of the snail-spread environments were located in the hilly region after flooding; furthermore, the area ratio of the snail spread, the percentage of occupied space in the frames with live snails, and the average density of live snails in the hilly region were significantly higher than those in the lake region. It is suggested that hilly areas are more susceptible to flooding than lake areas; therefore, the risk of snails spreading in hilly areas is greater than in other areas and, due to the complexity of this environment and its proximity to villages, residents and livestock are in contact with water more frequently due to production or living; thus, they are more at risk. However, the spread area of snails in the lake areas reached 13.88 km^2^ after flooding, which was nearly five times that of the hilly areas, mainly because of the flat terrain and single vegetation; the areas with snails in the lake region increased sharply after flooding. In the hilly region, the distribution of Oncomelania snails was relatively isolated, often appearing as small units that were not connected to each other along the water system, with small areas of snails, spotty patterns of distribution, and complex and diverse environments [29].

New snail areas often appear in hilly regions, which are closely related to the complex environments and crisscross water systems. Floods are likely to cause oncomelanid snail colonization, breeding, and reproduction in adjacent environments, to which water systems are connected (or interlinked). In lake regions, flooding can easily lead to the reappearance of or increase in snails due to flat beach areas. In addition, the area where snails were found for the first time was in the adjacent non-endemic zones that were not frequently included in the annual snail-survey plan and so were easily overlooked by investigators. They could only be detected when the density of snails was high, with a high occurrence rate in the snail frames. Snail-reproduction or -expansion environments often appear in historical snail environments, which are included in the local snail-monitoring range all year round. Therefore, these environments can often be discovered in time, so the density of live snails and the amount of space occupied in the frames by live snails is low.

This study showed that the occurrence area and the magnitude of flooding determined the spatial distribution and scope of snail spread [30,31,32,33,34]. From 2016 to 2019, in Jiangxi Province, the flood disasters were mainly concentrated in the hilly areas of northeastern Jiangxi and in the western section of Yangtze River. The flooded areas reflected the spatial distribution of the snail spread, which mainly encompassed the hills of northeastern Jiangxi, Yangtze River, and the watercourse of Poyang Lake into Yangtze River. No snail spread occurred in the main lake area of Poyang Lake.

In 2020, a catastrophic flood exceeding that which took place in 1998 occurred in the Poyang Lake basin, covering almost all of the snail-infected areas in Jiangxi Province. The snail-survey data in 2021 showed that snail spread occurred not only in the hilly areas of northeastern Jiangxi and Yangtze River, but also in the upper elevation area of the beach of the main lake area of Poyang Lake. This may have been related to the migration mode of oncomelanid snails, which can be dispersed through suspended mass, transported mass or by water surface-transport, which is affected by the environmental water depth, flow velocity, and floating objects [34].

For example, local floods can easily cause snails in hilly areas and on the Yangtze River beach to spread in the mass-transfer mode downstream along the surrounding water system. Moreover, the constant water level of Poyang Lake makes it difficult for snails to spread to the upper elevation of the beach with the movement of suspended sediment, but when floods recede, the snails can be moved with the suspended sediment to a lower elevation of the beach. Long-term super-warming water levels can cause oncomelanid snails to move upward with suspended sediments to the higher elevation of marshlands in the lake area [35].

At the same time, the snail spread has no obvious relationship with simple annual hydrological characteristics, but it is closely related to flooding in regional areas, especially water-level changes. This study showed that the diffusion distribution of snails from 2016 to 2020 was consistent with the distribution of local flood disasters. Although 2020 was a median water year, due to the basin-wide large flood in Poyang Lake in July, which broke the previous water-level record, the number of diffusion environments in 2021 was significantly higher than that in other years, accounting for 72.50% of the total in the five years covered by this study and 76.31% of the total diffusion area. This also suggests that there was a “lag effect” of the water regime on the diffusion of Oncomelania snails in that year, and recurrence, expansion, or the appearance of newly snail-infected environments occurred only one year after the flood disaster, which was consistent with previous reports [36,37,38,39].

The survey data showed that different flood-invasion types have different effects on snail habitats. Rainstorm flooding and storm-debris flow mainly affect snail spread in hills, and the mean density of live snails and the percentage of occupied space in frames with live snails were higher than those in the submerged lake. The main reasons for these observations were as follows. First, a sudden catastrophic flood could have caused the snails to migrate long distances downstream with the water flow in mass-transfer mode, leading to the snails’ redistribution in hills, ditches, paddy fields, and river beaches. Second, the storm-debris flow could easily have caused waterlogging and encouraged the migration and diffusion of snails or young snails to suitable surrounding environments in the form of suspended matter, and even led to the emergence of new snail spots in non-endemic villages.

For example, the newly discovered snail distribution, found in 2021, in a previously snail-free village (Sangyuan Village) in Yushan County was due to the catastrophic flooding disaster in 2020. Third, the groundwater and karst caves are rich in the endemic area of Jiangxi. The groundwater in these karst caves is swollen due to the large basin-wide floods, resulting in residual snails drifting with the groundwater flow, in the form of surface transportation, to the ground to recolonize and reproduce. For example, snails were captured 51 years after they were eradicated from an area in Licun village, Wuyuan County.

Lake submersion is the main factor affecting snail spread in the Poyang Lake area. Studies have shown that the water level affects the elevation distribution of snails in this type of environment. The duration of high water levels affects the range and distance of snail diffusion, and the velocity and direction of the water flow affect the snails’ spreading modes and destinations [31,35].

In the flood season, due to the double impact of the incoming water from the five rivers and the top support of Yangtze River, the water level of Poyang Lake’s backflow rises rapidly. Young or adult snails, along with floating objects, are affected by the water back-flow and reach the upper elevation of the beach, which has no snails. The higher the water level, the greater the distance of the lake’s top-supported back-flow, resulting in higher elevation and, consequently, the distribution of the highest snail line in the lake area. For example, a total of 15 snail-recurrence environments in the main lake area of Poyang Lake were discovered in 2021, all of which were distributed on the north shore of Poyang Lake and at the tail of Ganjiang River, where the elevation is above 16.1 m.

In addition, the young snails or adult snails drift downstream, transported along the water surface during the rapid decline of the lake water after flooding, and the consequent lower water level causes the snails to spread to the lower elevations of the beaches. For example, in 2019 and 2020, there were three diffusion environments at the mouth of Poyang Lake, with an elevation below 12.5 m, indicating that some water-regime factors, such as the annual water-level changes, the periodic inundation, and the dew duration of the marshland, can also determine the nature of the snail diffusion, density, and distribution area on the submerged beaches of the lake.

Schistosomiasis control is a complex systematic project, and flood disasters make this prevention and control work more complex and arduous. Numerous studies have shown that the first-time occurrence and reoccurrence areas of snails after flooding disasters can reach 2.16 and 2.56 times those in normal years, respectively; they may affect the snail distribution over the subsequent 3–5 years, and may have a continuous impact on the prevalence of schistosomiasis [40,41,42]. Following 70 years of prevention and control, no newly infected individuals or livestock have been detected in Jiangxi Province since 2020. The number of existing schistosomiasis patients is no higher than 6000, and the schistosomiasis epidemic is at its lowest level in history. However, susceptible individuals, such as travelers and anglers, are still at risk, and awareness of prevention and control is quite weak, posing a potential risk of schistosomiasis epidemics in areas to which snails have spread. Therefore, in addition to strengthening health education and disease surveillance for susceptible populations, it is truly necessary to strengthen snail monitoring in disaster-affected areas to eliminate snail distribution and spread in a timely fashion, to formulate precise mollusk-control programs according to the local conditions, and deal with newly discovered snail spots as soon as possible, so as to prevent the further spread of snails caused by floods.

In hilly regions, in addition to strengthening the measures of snail control, coordination and cooperation between departments should be strengthened. Projects, such as the structural adjustment of the planting and breeding industries, the transformation of high-standard fertile land, the restoration of barren fields and replanting, and environmental improvements should be given priority to cover snail areas and, consequently, completely change the snail-infected environment. In lake regions, the focus should be on strengthening the implementation of infection-source-control measures, such as prohibiting grazing in snail-infected marshland, replacing cattle with machines, carrying out drug spraying to kill snails or larvae in high-risk areas with schistosomes, and promptly repairing water-damaged projects and facilities to prevent the post-disaster rebound of endemic schistosomiasis [25,28].

## 5. Conclusions

Snail spread is one of the main reasons for schistosomiasis transmission, and flooding disasters are the main factors causing snail spread. This study has shown that the dispersal of oncomelanid snails after floods has a certain “lag effect,” and that simple annual changes in hydrological characteristics have no obvious effect on the dispersal of Oncomelania or on the snail density in the dispersal environment, which is more closely related to local floods. Hilly areas are more susceptible to floods than the lakes, the number of diffusion environments and the snail-diffusion-area ratio in these areas are greater than in lake areas, and the risk of snail proliferation is also much higher than in lake areas. Therefore, in the future, it is necessary to strengthen the monitoring of snail spread after flooding, to take control measures according to the local conditions to prevent snail spread, and to further strengthen the implementation of infection-source-control measures, such as the prohibition of grazing in snail-infected areas and the use of machines instead of bovines to prevent the rebounding of the schistosomiasis endemic. This research is of important guiding significance for guiding China’s post-flood snail monitoring and to formulate snail-diffusion-control measures in the future.

## Figures and Tables

**Table 1:** Spreading areas of Oncomelania hupensis in different endemic regions from 2017 to 2021.

Year	Hilly and Mountainous Region			Marshland and Lake Region		
	Actual Snail-Infested Area Total (km^2^)	Area with Snails Detected for the First Time (km^2^)	Proportion (%)	Actual Snail Infested-Area Total (km^2^)	Area with Snails Detected for the First Time (km^2^)	Proportion (%)
2017	24.31	0.02	0.09	809.28	0.59	0.07
2018	24.30	0	0	809.83	0	0
2019	25.22	0.31	1.24	810.08	0.52	0.06
2020	25.29	0.78	3.08	810.63	0.55	0.07
2021	27.41	1.81	6.59	821.97	12.21	1.49

**Table 2:** The composition of Oncomelania hupensis’ spreading properties.

Property	Number of Diffusion Environments		Area (km^2^)	The Mean Density of Live Snails (No./0.1 m^2^)	The Space Occupied by Live Snails (%)
	Hilly and Mountainous Region	Marshland and Lake Region			
Recurrence	39	23	14.19	0.20	11.74
Expansion	33	5	2.35	0.62	33.90
First-time occurrence	14	0	0.12	0.53	37.19
Total	86	28	16.66	0.46	22.92

**Table 3:** Precipitation and the hydrological characteristics in Jiangxi Province from 2016 to 2020.

Year	Average Precipitation in the Province (mm)	Comparison with the Multi-Year Average (mm)	Type
2016	1997.0	21.9	wet year
2017	1637.2	5.9	median water year
2018	1129.9	−26.9	dry year
2019	2032.7	31.5	wet year
2020	1666.7	7.8	median water year

**Table 4:** Effect of different types of flood invasion on snail spread.

Type	Number of Diffusion Environments		Area (km^2^)	The Mean Density of Live Snails (No./0.1 m^2^)	The Space Occupied by Live Snails (%)
	Hilly and Mountainous Regions	Marshland and Lake Regions			
Storm flood	66	10	4.70	0.58	36.48
Storm-debris flow	20	0	2.55	0.23	12.77
Submerged lake	0	18	11.71	0.04	2.15
Total	86	28	16.66	0.46	22.92

## Data Availability

The data used in the study are available from the corresponding author upon reasonable request.
